# Data demonstrating the Finnish wood pellet industry and future perspectives

**DOI:** 10.1016/j.dib.2016.11.018

**Published:** 2016-11-12

**Authors:** Svetlana Proskurina, Jussi Heinimö, Mirja Mikkilä, Esa Vakkilainen

**Affiliations:** a Lappeenranta University of Technology, Skinnarilankatu 34, 53850 Lappeenranta, Finland; b Mikkeli Development Miksei Ltd, Mikkeli, Finland

## Abstract

This article contains data related to the research article entitled “A survey analysis of the wood pellet industry in Finland: Future perspectives" (S. Proskurina, E. Alakangas, J. Heinimö, M. Mikkilä, E. Vakkilainen, 2016) [1]. The dataset include information about the importance of wood pellets on the global bioenergy development and role of wood pellets on the Finnish bioenergy development. Data leads to an expansion of knowledge and discoveries of new possibility for wood pellets industry analysis.

**Specifications Table**

**Table:** 

Subject area	*Energy*
More specific subject area	*Wood pellets*
Type of data	*Text file*
How data was acquired	*Survey*
Data format	*Raw, analyzed*
Experimental factors	*The questionnaire*
Experimental features	*The results of a survey of 58 wood pellet experts*
Data source location	*n/a*
Data accessibility	*Data available within this article*

**Value of the data**

• The data show opportunities and challenges of Finnish wood pellets development.
• It allows predicting the future wood pellets markets in Finland.
• Data helps to make future actions and managements for improving the development of wood pellets within the country and outside.
• Data leads to an expansion of knowledge and discoveries of new possibility for wood pellets industry analysis.
• The data can be used to analyze the wood pellets markets for other countries, similar opportunities and challenges can exist in many other European countries.

## 1 Data

The shared data describes the role of wood pellets in bioenergy development in Finland [1]. Data collects information about the current status and future perspectives of the Finnish wood pellet industry. Contents include literature review of global wood pellets market, advantages of wood pellets as fuels, characteristics of Finnish wood pellet markets. About 60 experts in wood pellets industry in and outside Finland express own view about the current situation of wood pellet industry and its future perspective. Appendix 1. More data can be funded in research article by Proskurina et al., 2016 [1].

## 2 Materials and methods

The data analysis of the Finnish wood pellet industry was done using a qualitative approach. It based on literature reviews combined with the results of a questionnaire, which is a typical qualitative research method [2].

## Footnotes

Appendix A Supplementary data associated with this article can be found in the online version at doi:10.1016/j.dib.2016.11.018.
