# Supplementary material for: Data demonstrating the Finnish wood pellet industry and future perspectives
Source: Data Brief. 2016 Nov 12;10:41–3. doi: 10.1016/j.dib.2016.11.018 (PMC5137334; doi:10.1016/j.dib.2016.11.018)
Supplement: Supplementary file 2 — Supplementary material [file mmc2.docx]

Data about wood pellets in Finland

Opinions about factors, which are stimulating/inhibiting development of the Finnish wood, pellet industry:

- Government support of wood pellet industry, support from EU.

- Development of domestic consumption and domestic demand

- Domestic policies, taxation, support/allowances

- Rising prices of other fuels, oil-heating tradition

- Low price of electricity and oil

- Strong availability of cheaper types of biofuel

- Availability of reasonably priced raw material from sawmill business

- Price and taxation of fossil fuels

- Emission limits for heavy-duty oil

- Price of EU emissions allowances (CO_2_),

- Lack of nation-wide full service providers, especially of residential market.

- Common economic trends globally affecting the sawmilling volumes.

- Speared of suitable boilers

- Marketing

- Establishing "right" connections and networks

- "Competitive pellets" from other countries,

- High price level of raw material and wood pellets

- Logistics, transportation cost, infrastructure for long distance supply chains

- Private initiative

- Competition with other forest industries

- Investments in pellet-boilers

- Competitiveness of domestic production compared to other producers that operate in the same international market

- Consumer guidance

- Strong wood log etc.…
