# Supplementary material for: Data demonstrating the Finnish wood pellet industry and future perspectives
Source: Data Brief. 2016 Nov 12;10:41–3. doi: 10.1016/j.dib.2016.11.018 (PMC5137334; doi:10.1016/j.dib.2016.11.018)
Supplement: Supplementary file 1 — Supplementary material [file mmc1.docx]

Dear Editor,

This is to confirm that there is **no conflicts of interest** involved with the manuscript entitled “A survey analysis of the wood pellet industry in Finland: Future perspectives”.

Thank you very much for your consideration.

Best Regards,

Svetlana Proskurina
